# Exploration of the Genetic Diversity of Solina Wheat and Its Implication for Grain Quality

**DOI:** 10.3390/plants11091170

**Published:** 2022-04-26

**Authors:** Riccardo De Flaviis, Giorgio Tumino, Valeria Terzi, Caterina Morcia, Veronica Santarelli, Giampiero Sacchetti, Dino Mastrocola

**Affiliations:** 1 Faculty of Bioscience and Technology for Food, Agriculture and Environment, University of Teramo, Via R. Balzarini 1, 64100 Teramo, Italy; rdeflaviis@unite.it (R.D.F.); vsantarelli@unite.it (V.S.); dmastrocola@unite.it (D.M.); 2 Plant Breeding, Wageningen University and Research, Droevendaalsesteeg 1, 6708 PB Wageningen, The Netherlands; giorgio.tumino@wur.nl; 3 Consiglio per la Ricerca in Agricoltura e l’Analisi dell’Economia Agraria—Centro di Ricerca Genomica e Bioinformatica (CREA-GB), Via San Protaso 302, 29017 Fiorenzuola d’Arda, Italy; valeria.terzi@crea.gov.it (V.T.); caterina.morcia@crea.gov.it (C.M.)

**Keywords:** landrace, local germplasm, single nucleotide polymorphism, DArTseq markers, population structure, commercial quality, genetic diversity, wheat relationships

## Abstract

Different Solina wheat accessions (*n* = 24) collected in the Abruzzo region (Italy) were studied using 45,000 SNP markers generated from the DarTseq platform. The structure of genetic data was analyzed by Principal Component Analysis and Hierarchical Cluster analysis that revealed the existence of two main clusters (Clu1 and Clu2) characterized by samples with different geographical origin. The Solina genetic dataset was further merged and analyzed with a public genetic one provided by CIMMYT containing 25,963 genotypes from all over the world. The Solina accessions occupied a vast space, thus confirming a high heterogeneity of this landrace that, nevertheless, is considerably unique and placed quite far from other clusters. Clu1 and Clu2 divergence were clearly visible. Solina clusters were genetically closer to landraces from Turkey and the central fertile crescent than to the Italian genotypes present in the dataset. Selected commercial quality traits of accessions of the two Solina clusters were analyzed (yield, thousand kernel weight, test weight, and protein content), and significant differences were found between clusters. The results of this investigation did not highlight any relationships of Solina with Italian genotypes, and confirmed its wide genetic diversity by permitting to identify two genetic groups with distinct origin and quality traits.

## 1. Introduction

Solina is a historically cultivated landrace in Abruzzo (central Italy) and strongly linked with the territory and culture of mountain rural areas. It is not clear from where and when the Solina came out. It is surely possible to affirm that Solina is sown at least since the sixteenth century when it is mentioned in some notary deeds of sale and purchase stipulated at the Lanciano Fair [1].

From an agronomic point of view, Solina is very rustic, well suited to poor soils and those consisting of stone, very resistant to cold weather (it can resist for a long time covered by snow), and to the spring–summer drought, of limited productivity (on average 2 t/ha) but offering constant production; these traits favored its wide use on a territorial scale [2]. Seed circulation for such a long time span with mixing and selection, adjuvated by natural selection, could have promoted the recombination between different Solina genotypes and the fixation of neutral and adaptive mutations [3]. Despite its diversity, Solina is in fact a considerable Distinct and Stable landrace since it showed a phenotypic diverge similar to that of pure lines [4].

In the early twentieth century, Solina wheat has been extensively used in breeding programs carried out in Italy [5] but scientific interest has gradually grown only in recent years, as demonstrated by several recent publications [2,3,4,5,6,7,8,9,10,11,12]. Given the importance that such landrace constitutes both from a historical and economic standpoint [2,13,14], in the year 2007, it was inserted between the target landraces in a European study aimed at seed conservation, breeding, and production [3,4,9], thus it is of utmost importance to collect and genotype it.

The Green Revolution (1940–1960) allowed the doubling of wheat production, responding to the doubling of the world population in the last 40 years. The spread of highly productive varieties, together with the massive use of fertilizers and crop protection products, permitted a spectacular increase in agricultural production, in particular wheat and rice. At an environmental level, this intensive agriculture has been considered, in accordance with “the Borlaug hypothesis”, a tool to conserve the biodiversity of ecosystems, allowing land sparing in favor of natural environments [15]. 

Green Revolution and, in general, modern breeding, are involved in the erosion of genetic diversity in wheat: the use of a limited number of parental genotypes in breeding reduced the genetic basis of this crop, causing a genetic bottleneck. The genetic heritage represented by many old cultivars and landraces was put aside in favor of a few High Yielding Varieties (HYV), which were disease- and lodging-resistant, highly responsive to input, and characterized by Distinctness, Uniformity and Stability (DUS). Since modern cultivars were implemented primarily under modern agronomic practices using high agricultural inputs, several landraces (traditional crop varieties) remained protected and continuously cultivated in marginal areas, thanks also to their high adaptability in unfavorable growing conditions (e.g., wet, cold soils, high altitudes, and poor soils) [16].

In this context, landrace seeds’ management became typically an in situ on-farm conservation conducted by local farmers themselves, and at the same time, allowed to avoid genetic erosion and to obtain new alleles, deriving from casual mutation or from other neighboring populations [17]. Genetic diversity was preserved and increased by traditional and empirical agronomic practices used by local farmers, and nowadays, many scientific efforts are aimed to understand and exploit it [18]. 

In order to unravel the genetic structure and breeding history lying behind landraces, a genome-wide approach is needed; thanks to modern high-throughput technologies, such as DarTseq (Diversity Arrays Technology sequence), researchers can efficiently genotype thousands of genomic loci in parallel with a relative low cost [19]. Recently, DarTseq has been applied in complex polyploid crops, such as wheat, to find unexplored loci in landraces and elite wheats [20,21,22,23,24]. Using this technology, Sansaloni and co-workers [25] analyzed the genetic structure of 56,342 hexaploid wheat accessions with 85,531 single nucleotide polymorphism (SNP) markers, proving that the modern cultivars occupied only a little portion of the total genetic diversity space; thus landraces show a high unexplored biodiversity worthwhile of being studied and protected. 

In this context, important is the work of Khan and co-workers [3] who studied European populations (in situ and ex situ varieties) of common wheat. Using 41 SNP markers located in neutral regions, the authors found that the genetic complexity increases from modern varieties to on-farm landraces, and that Solina wheat (Italic landrace) shows the highest genetic and haplotypic diversity among few European varieties [3]. The genetic diversity of Solina is difficult to explain for a self-pollinated species; the authors supposed that a very large effective size, caused by an on-farm collective management, could have led to this phenomenon. Seed circulation with mixing and selection, adjuvated by natural selection, had promoted recombination between different genotypes and the fixation of neutral and adaptive mutations. 

Nevertheless, the pioneering studies above reported presented two major limitations: the number of SNPs analyzed and the number of accessions collected per landrace (farmer populations). According to Negri and co-workers [17], “on-farm conservation should be reformulated as the management of genetic diversity of locally developed crop varieties (landraces) by farmers within their own agricultural, horticultural or agri-silvicultural systems”. Agricultural systems, understood as the set of agronomic practices and soil characteristics, can be different among different farms, potentially impacting on the genetic variability of the landrace. As a matter of fact, most studies in the field of on-farm landrace genetic characterization are more focused on within-accession diversity than in the between-farmer populations diversity. This study attempted to overcome these weaknesses, focusing on Solina wheat (*Triticum aestivum*, L.) and increasing the number of accessions collected and SNPs analyzed. Solina accessions, in situ conserved by different farmers, were characterized for both intra- and inter-farmer accessions genetic diversity, and also evaluated in relation with some quality parameters.

Moreover, in order to identify any genetic relationships within a vast common wheat germplasm and therefore shed light on the Solina history, the Solina genetic dataset was merged with a public genetic one (CIMMYT) obtained by 25,963 different common wheat genotypes from all over the world and further analyzed. To the authors’ knowledge this is the first attempt to merge a landrace genotyping dataset with the international CIMMYT dataset to gain information on landrace genetic variability and relationships.

## 2. Materials and Methods

### 2.1. Grain Samples

**Figure 1:**
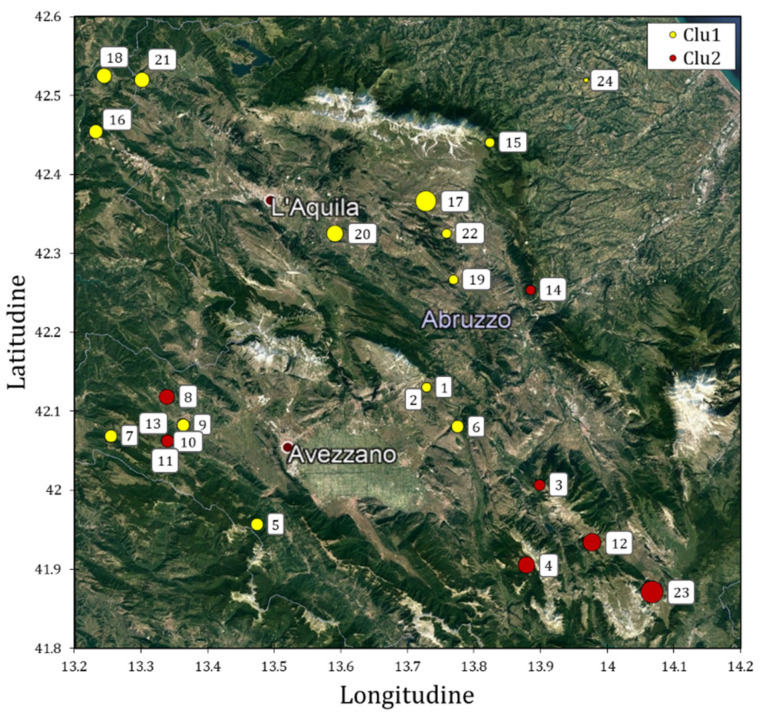
Geographical location of 24 Solina accessions collected in 2018 in the Abruzzo region colored on the basis of their genetic clusters identified by DArT markers analysis (see Section 3 and Section 4). The diameters of yellow and red circles are correlated to the altitude of the site of seed collection. Dark red dots with a white border represent the most important cities of the area.

Twenty-four accessions of Solina wheat were collected in several locations, representative of Solina cultivation area in the Abruzzo region (Supplementary Table S1). Figure 1 shows the Solina IDs projected on the Abruzzo region map, based on the collection sites.

The sampling was conducted in order to obtain the greatest number of accessions representative of the typical areal of Solina cultivation. The sites were distributed mainly in the province of L’Aquila (only two accessions were sampled in the province of Pescara); their average elevation was 755 ± 263 m above sea level and they were characterized by different types of pedo-climatic conditions, distinctives of the Abruzzo region.

The accessions were sampled from grains harvested in the year 2018 in different farms in which Solina wheat was cultivated for at least ten years under organic management. In order to evaluate the genetic variability within accessions and among accessions, four single seeds and one seed bulk (50 seeds) were sampled per accession and named A, B, C, D, and E, respectively. 

Seeds of accessions 2 and 3 (Supplementary Table S1) supplied by Az. Agricola Cipolla, (Castelvecchio Subequo, Italy) and Az. Agricola De Santis (Introdacqua, Italy), respectively, were used for field trials conducted in randomized plots in the Abruzzo region (Italy). The experimental fields were located in two different farms coded as F1 and F2 sited in Corropoli (42°49′ N, 13°51′ E, 70 m a.s.l.) and Castelvecchio Subequo (42°07′ N, 13°44′ E, 500 m a.s.l), respectively, that presented different soil and climatic characteristics. The two accessions were cultivated in the 2018/2019 and 2019/2020 seasons. The sowing was done between the end of October and the beginning of December 2018 and 2019, in a 500 m length plot area. The seeding rate was of 250 kg/ha, under rainfed condition. Plot width was 50 m and the distance between plots was 1.5 m. Plants were grown under the same organic conditions, and no pesticides (bio- or chemical) and fertilizers (organic or chemical) were used. All the grains were harvested between the starting and the middle of July 2019 and 2020, depending on the elevation of the fields. The phytosanitary quality of crops was periodically controlled during the two years of cultivation and plants were healthy field-grown plants. The productivity of Solina wheat at 70 and 500 m a.s.l. was of about 20 q/ha in both years and was similar to those observed in the area of cultivation under organic regimes.

### 2.2. Genotyping

The samples were pre-treated following the instructions provided by Diversity Arrays Technology (DArT) Pty. Ltd. (http://www.diversityarrays.com, accessed on 1 December 2021, Canberra, Australia): single seeds were pounded separately avoiding DNA cross-contamination, and contemporary 50 seeds for every accession were milled; 96 pounded Seeds and 24 milled Bulk (20 mg per accession) were carefully placed in two 96-well plates and shipped with silica gels to DArT laboratories for SNP analysis (DNA extraction, DArTseq library preparation, sequencing on Illumina Hiseq2500/Novaseq using 2.5 million reads per sample, marker data extraction, and alignment to publicly accessible sequence of wheat (Wheat_ChineseSpring20 and Wheat_ConsensusMap_version_4)); 45,055 SNP markers were obtained and used for further statistical analyses.

### 2.3. Solina Dataset Pre-Processing and Filtering

In order to ease data structure representation and statistical elaboration, the DArT dataset (120 samples × 45,055 markers) was rearranged as trinary values (allelic dosage of alternate alleles) as follows: (i) “0” when only the reference allele homozygote is present; (ii) “1” when both the alleles are present (heterozygosis); (iii) “2” when only the SNP allele homozygote is present; (iv) “null” when both the alleles are absent (absence of fragment with SNP in genomic representation); “null” were considered as missing.

Subsequently, several quality SNP filters were applied to check uninformative or misinformative markers or genotypes: (i) exclusion of SNP markers and genotypes with more than 25% missing values; (ii) exclusion of SNP markers with less than 5% of Minor Allelic Frequency (MAF).

Remaining missing values were imputed by using the marker means.

### 2.4. Public CIMMYT Dataset Importing, Pre-Processing, and Filtering

The study of [25], implemented by CIMMYT (Centro Internacional de Mejoramiento de Maíz y Trigo) in collaboration with ICARDA, INIFAP, NIAB, EI, and DArT is a recent milestone in wheat genetics. Their huge amount of results are publicly available at https://data.cimmyt.org/ (accessed on 10 January 2020) and were downloaded and rearranged (see Section 2.3) to be merged with the single Seed dataset. From the hexaploid CIMMYT dataset were imported 9670 SNP markers (only those in common with the single Seeds dataset, using both *AlleleID* and *AlleleSequence* as marker identificators) and 25,963 genotypes of cultivars and landraces of *Triticum aestivum* L. *aestivum*, which in combination with 96 single Seeds produced a unique matrix of 26,059 genotypes with trinary values. Since significant differences in heterozygosis have been seen between CIMMYT and Solina genotypes, stricter filters were used: (i) exclusion of SNP markers with more than 10% missing values; (ii) exclusion of genotypes with more than 20% missing values; (iii) exclusion of genotypes with more than 10% heterozygosis; (iv) exclusion of SNP markers with less than 5% of MAF.

### 2.5. Commercial Quality

Phenotyping of three commercial quality parameters was done for every accession in duplicate. The total nitrogen content (NC) was determined using the Kjeldahl method (AACC 46-12.01): the sample is digested in sulfuric acid, ammonia is distilled, and then the distillate is titrated with hydrochloric acid. A conversion factor of 5.7 was used for wheat. Test weight (TW) and thousand-kernel weight (TKW) were analyzed after removing all impurities. Moisture content was also analyzed (AACC 44-15.02).

### 2.6. Statistical Analysis

Statistical analyses of genetic data were carried out using R 4.0.4 (R Core Team 2020) and several specific packages. Missing value replacement was done using *na.mean* function (R package *imputeTS*). SNPs density plot were obtained thanks to *CMplot* function (R package *CMplot*). Principal Component Analysis (PCA) based on covariance matrix was computed by the *prcomp* function (R package *stats*), whereas Hierarchical Clustering Analysis (HCA) was performed using Euclidean distances and average-linkage agglomeration method implemented in the *hclust* function (R package *stats*). Moreover, PC1 scores (obtained with single seed dataset) were used in multiple linear regression (MLR) with geographic coordinates (latitude longitude and altitude) as explanatory variables; thus β coefficients were calculated in order to compare the relative weights of the variables.

Statistical analysis of phenotypic data was performed using XLSTAT 2021 software (Addinsoft, Paris, France). Mixed nested ANOVA was carried out on phenotypic data from the 24 accessions of Table S1 by considering the Clu1/Clu2 as fixed effect and the farm of collection as the random nested effect. Repeated measurement ANOVA was carried out on data of accessions 2 and 3 (representative of Clu1 and Clu2, respectively) cultivated in farm F1 and F2 for two consecutive years, and plot was used as random effect.

## 3. Results

### 3.1. Solina Datasets: DArTseq Marker Characteristics

DArTseq analysis permitted to detect approximately 45 K SNPs for each of the 120 Solina samples investigated. Original Solina dataset was initially divided into Bulk (24 genotypes) and single Seed (96 genotypes) matrices, and were studied always separately. Bulk genotypes may be comparable to a genetic superimposition of 50 seeds, tested to evaluate a larger sample size, and so, the within-accession variability [26]. After the application of several quality check filters, the datasets were reduced to 23,741 SNP markers and 24 genotypes for Bulk dataset, and to 15,959 SNP markers and 93 genotypes for single Seed dataset. Three seeds from three different accessions (20A, 21D, and 24A) were removed from the single Seed dataset since they had more than 25% of missing values. On the other hand, 13,181 and 13,719 markers for Bulk and single Seeds datasets were considered rare alleles and discarded (MAF < 5%). On average, the filtered datasets had 6.5% and 6.7% of null, and 40.4% and 12.8% of heterozygosis for Bulk and single Seeds, respectively (Supplementary Figure S1). Only one Bulk genotype (20E) had less than 25% of heterozygosis whereas only two Seeds (17C and 18D) had more. The sample 20E was also the unique Bulk with more than 15% of missing values (but less than 25%) and, in the same way, the individuals 15A and 14A for single Seeds dataset. Among genomes, the B was always the most represented with 7650 and 5279 markers for Bulk and single Seed filtered datasets followed by A genome (7385 and 5130) and D genome (4277 and 3055) (Supplementary Table S2). However, several markers (4429 and 2495) were still unassigned to any of the 21 wheat chromosomes. The density plots in Supplementary Figure S2a,b confirmed the wide genome representation for each chromosome in both datasets. The highest SNPs density was always observed in chromosome terminations whereas the lowest was near the centromeres, as already observed by [22]. A clear difference is present between single Seed and Bulk in marker density, as a consequence of different number of markers selected by filters. The homoeologous chromosomes of the fourth group were the least represented and, in fact, only the chromosomes 4D had less than 300 markers for both datasets (Supplementary Table S2).

### 3.2. Solina Population Structure

**Figure 2:**
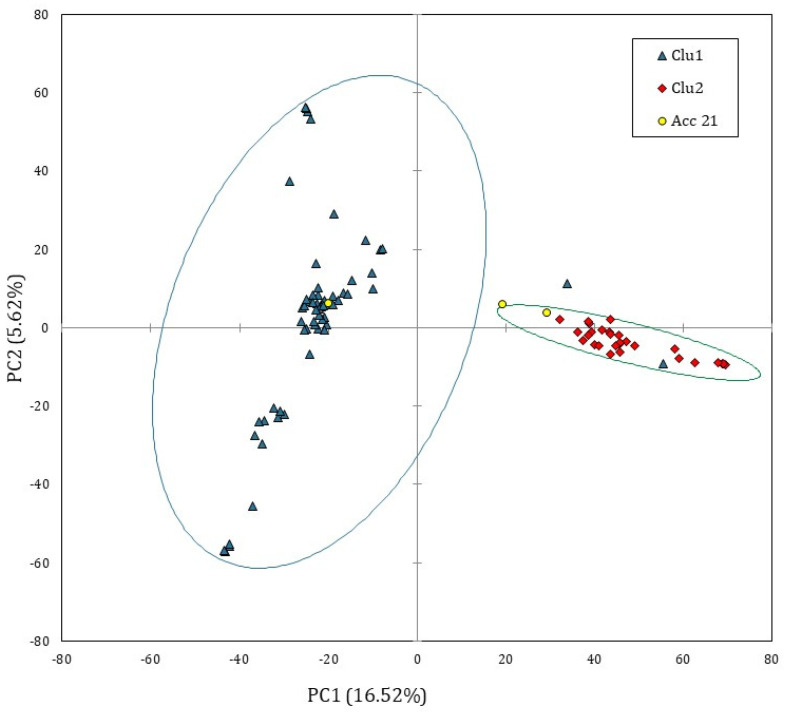
PCA score plot computed by using single Seed dataset (15,959 SNP markers and 93 genotypes). The ellipsis indicate the 95% confidence limit for each Solina cluster. The accession 21 was considered an outlier.

In order to explore the genomic structure of Solina accessions, a PCA based on covariance matrix was computed only on single Seed dataset; since *prcomp* function is not able to process missing values, they were substituted by marker means. Figure 2 shows the score plot of 93 Solina seeds using the first two principal components (PCs) which explained the 22.14% of the total variance, 16.52% along the PC1 and 5.62% along the PC2. 

**Figure 3:**
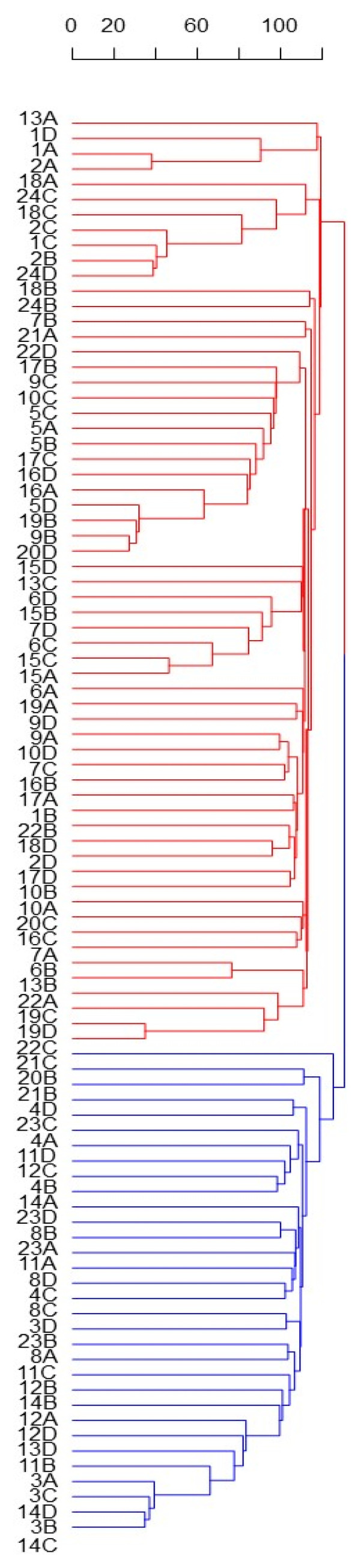
HCA based on Euclidean distances and average-linkage agglomeration method computed by using single Seed dataset (15,959 SNP markers and 93 genotypes). Labels indicate the number ID of the 24 Solina accessions, and letters correspond to the four different seeds (A, B, C, D) analyzed. Two Solina clusters were highlighted by different colors.

Despite the small, explained variance, the PC1 permitted to identify two main and distinct Solina clusters (Clu1 and Clu2), as highlighted by confidential ellipses in Figure 2. Clu1 is composed by 16 farmer accessions (1, 2, 5, 6, 7, 9, 10, 13, 15, 16, 17, 18, 19, 20, 22, and 24), while in Clu2 by 7 (3, 4, 8, 11, 12, 14, 23), only two seeds of Clu1 from two different accessions, 20B and 13D, deviate from this classification. The accession 21 remained ambiguous; indeed, one seed (21C) was located exactly between the two clusters whereas another seed (21B) was similar to other genotypes in Clu2. Differently, PC2 mostly described genetic variation within the Clu1 without showing further net separation of samples on the basis of their genetic traits. Similar results were obtained with HCA based on Euclidean distances and average-linkage agglomeration method, supporting the two Solina clusters hypothesis. Moreover, Euclidean distances revealed considerable similarities between accessions (at least within Clu1 or Clu2) but lower within accessions, as shown by the dendrogram in Figure 3.

PC1 scores were further used in multiple linear regression (MLR) using latitude, longitude, and altitude (Supplementary Table S1) as explanatory variables. The model showed an R^2^_adj._ value of 0.231 (*p* < 0.001), and significant β-coefficients (*p* < 0.05) were found with the following values: −0.31, 0.25, and 0.19. Clu2, characterized by positive scores in PC1, which tends to localize in the south-eastern area of Abruzzo region, that on average shows a higher altitude value.

### 3.3. CIMMYT/Solina Dataset: DArTseq Marker and Population Structure

To better analyze the genetics of Solina accessions in an international and multi-varieties context, a public SNP database provided by CIMMYT [25] was imported; 9670 markers and 26,059 genotypes of common wheat assemble the CIMMYT/Solina merged dataset that, after several and more strict filters, was reduced to 3842 markers and 24,868 genotypes of which 91 were Solina seeds. Three Solina seeds (20A, 21D, and24A) were discarded once again (see Section 3.2), but also 260 CIMMYT genotypes with more than 20% of missing values were removed. Regarding markers, 4872 with more than 10% of missing values and 956 with less than 5% of MAF were omitted from the final dataset. The heterozygosis filter was applied to uniform the heterozygosis between genotypes in the two datasets (initially higher in CIMMYT dataset), and to discard several genotypes that resulted very similar towards anyone else. Thus, 928 CIMMYT genotypes and two Solina seeds (18D and 17C) were removed since they had more than 10% of heterozygosis. The chromosomal distributions of markers follow the one previously described for Solina datasets, although with a reduced number of markers (Supplementary Table S2 and Supplementary Figure S2c). 

The filtered merged dataset is constituted of 3058 cultivars and 21,719 landraces; the most represented nations were Mexico (10,614), Iran (3695), and China (2198). Only 28 genotypes were Italian. 

**Figure 4:**
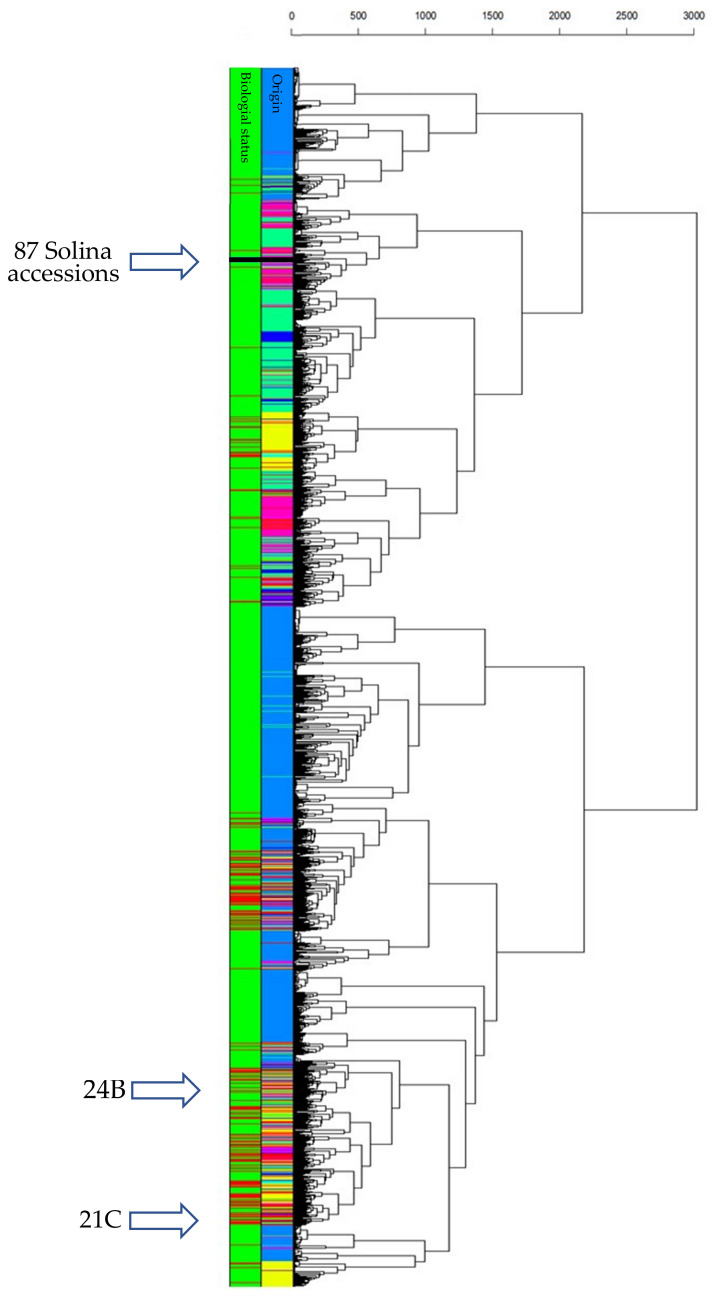
HCA based on Euclidean distances and average-linkage agglomeration method computed by using CIMMYT/Solina dataset (3842 markers and 24,868 genotypes of which 91 were Solina seeds). Two rows of labels indicate wheat origin (95 colors for each country: dodger blue for Mexico, spring green for Iran, deep pink for Turkey, yellow for China, black for Solina) and biological status (green for CIMMYT landraces; red for CIMMYT cultivars; black for Solina genotypes).

To understand how the Solina accessions related with CIMMYT populations, PCA and HCA were computed. Since this dataset was larger than previous ones, a clearer 3D score plot was depicted and shown in Supplementary Figure S3. The first three PCs explained an overall variance of 19.32% (9.10%, 5.35%, and 4.87% for PC1, PC2, and PC3 respectively), showing 24,868 data points corresponding to just as many genotypes. Thanks to confidence 3D ellipsoids and colors point, an intuitive picture of the dataset can be made; the genetic structure depicted by PCA offered interesting information about worldwide genetic relationships and correlations of common wheat that were not further discussed in this work (for an in-depth discussion see [25]). However, landrace genetic diversity was considerably higher than cultivars that were located very close to each other within a portion of the space. The Solina accessions occupied a vast 3D space considerably unique, quite far from other clusters. Despite the limited number of markers, Clu1 and Clu2 divergence is clearly visible along the PC3, which is correlated to the protein content (r = 0.594) according to the CIMMYT phenotypic dataset. Going in-depth, a HCA provided further information about genotype linkages through Euclidean distances (Figure 4). In particular, average-linkage agglomeration method classified 89 Solina seeds together at 250 Euclidean distances, while two seeds of two different accessions (21C and 24B) diverge considerably starting from the first dendrogram branch and clustering near to a Switzerland genotype (Blanc-Precoce) and to Baionette I (a cultivar obtained by Nazareno Strampelli, breeding Rieti × Prince Albert), respectively. Clu1 and Clu2 seeds join together at 125 and 150 Euclidean distances, respectively, whereas the first CIMMYT genotypes are found at 410 Euclidean distances. Considering the first genetic outsiders, they are 545 landraces and 12 cultivars of which 294 (45%) genotypes are Turkish, 59 (11%) are Iranian, and 43 (8%) are Syrian. The geographic origin of 119 (21%) neighbor genotypes are unknown. No Italian genotypes are met in the dendrogram up to a Euclidean distance of 1710.

### 3.4. Commercial Quality Parameters

TWK, TW, and proteins were analyzed on the Solina sample set used for DArTseq analysis. Table 1 summarized the ANOVA results. Within the 24 Solina accessions, TWK and TW of the two Solina genetic clusters were significantly different (*p* < 0.05), even though the nested farm effect resulted always in the main effect (*p* < 0.001). No significant differences among clusters were observed for protein content which showed an extremely high variability (from 9.66 to 15.48% _d.b._).

The effect of genetic cluster on TWK and TW was confirmed by the in situ experiment carried out by comparing two Solina accessions cultivated in two different farms over two years, and this experiment highlighted also a significant difference in the protein content of the two accessions (Table 2). On the other hand, the main effect for experimental field data diverged for each variable: farm effect was predominantly in TWK whereas the year effect in TW. Plot was always not significative (*p* > 0.05). 

Clu1 was the cluster with the highest TWK and TW in both the sample set used for DArTseq analysis and the sample set obtained by the in situ experiment. In the latter experiment, Clu1 resulted in the genetic cluster with the highest value of protein content. This result reflects the highest score values of Clu1 along the PC3 calculated using the CIMMYT phenotypic dataset (see Section 3.3).

## 4. Discussion

The current study aimed at obtaining valuable information on Solina wheat, a historically Italian landrace, adapted to the mountain environment of central Italy; as a matter of fact, characterizing genetic population structures is a fundamental step towards landrace protection, conservation and proper utilization in breeding program, agronomic management, and product transformation. The challenge was to draw out useful results from a consistent genetic diversity, typical of landraces [3], which makes it difficult to set clear boundaries around these genotypes.

### 4.1. Population Structure Analysis

DArTseq analysis permitted to deeply investigate the Solina genome. As previously stated, wide genetic diversity is expected by local and historical landraces, and for this reason also, a bulk was considered in the analysis. The higher heterozygosis in Bulk dataset (Supplementary Figure S1) was the result of 50 milled individuals and of an evident genetic within-accession diversity; this confirms previous results reported by Khan and co-workers [3]. 

Even though the final datasets were significantly reduced, high-quality SNP markers have remained widely distributed abroad the three genomes, as shown in Supplementary Table S2. Genome D and the homoeologous chromosomes of the fourth group were characterized by the lowest number of markers; this result is in accordance with those of other authors [22,23,24] who analyzed Spanish, Iranian, and Afghan common wheat landraces with the same DArTseq approach. The limited diversity of D genome may be originated from the “recent” introgression occurred with *Aegilops tauschii* (2*n* = DD), the common wheat D-genome donor, and the relative genetic bottleneck [27]. 

Since SNP datasets are matrices with a great imbalance in variables/cases ratio, they were analyzed through an unsupervised multivariate approach to reduce dimensionality and better visualize huge datasets. A PCA based on covariance matrix and HCA based on average-linkage agglomeration method permitted to identify clearly two genetic clusters (Clu1 and Clu2). This is clearly shown in the score plot in Figure 2, where a clear separation is visible along the first two principal components, despite that the genotype 21, together with 20 and 13, in minor extent, was revealed to be very heterogenous. A possible explanation for this might be due to the seed management of that specific farmer. Accession 21 could be a landrace mixture of distinct local varieties, Solina and Baionetta included, and accessions 20 and 13, arguably, could be a mixture of Solina(s) Clu1 and Clu2. 

Recent works in this field demonstrated how geography was the main effect driving the genetic diversity [22,24], even though others did not find any geographical effect on genetics [20,23]. In this study, β-coefficients, calculated by MLR, clearly demonstrated how a geographic effect existed between Clu1 and Clu2. It is widely known that the environment, especially those with low resources and harsher climate, affects the genome of plants, and leads to genetic adaptation and stability [28]. As a consequence, the genetic difference observed between the two Solina clusters may be originated from different abiotic stresses due to different pedo-climatic characteristics of cultivation areas. 

Solina wheat is isolated from all the worldwide cultivars and landraces reported in the CIMMYT database so it could be considered as a distinct landrace but it shows a wide genetic variance, even stressing the results reported by Khan et al. [3] on a single accession.

In an attempt to clarify a possible historical origin of Solina clusters or to find an event that significantly affected its genetic diversity, an analysis of the first genetic outsiders was carried out and revealed that Solina is located neither near nor within the Italian genotypes cluster, and is very close to landraces from the central fertile crescent (mainly Turkish), which is the major center of wheat domestication.

It has been hypothesized that *Triticum aestivum* first arose in Turkey, in which earliest records dated 8600–7800 before present, have been found. However, it is even possible that several allopolyploid speciation processes occurred, still carrying several unanswered questions [29]. After speciation, a continuous process of diversification resulted in the development of numerous landraces with fine adaptation to different agroecological environments, and spreading of common wheat in humid and cold environments of Central and Northern Europe. 

The genetic similarity found among Solina and Fertile Crescent germplasm arises the question if Solina can be considered an allochthonous or an autochthonous landrace [30]. Zeven [31] defined autochthonous “*a landrace grown for a long period in the farming system concerned. As the environment changes annually and as the landrace becomes ‘contaminated’ with few genotypes of other landrace(s), or cultivar(s) it will continuously adapt itself*”. On the contrary, “*allochthonous landrace is an autochthonous landrace of a foreign region recently introduced into the region concerned. Depending on the number of generations of after growth and on the frequency of seed change, it may become an autochthonous landrace*”. Starting from the facts that Solina is an historical and dynamic population, with distinct identity, very well adapted to the Abruzzo agroecological conditions, it can be categorized as an Italian autochthonous landrace that still maintains strong genetic relationships with the landraces of the major ancient center of diversification of common wheat.

### 4.2. Commercial Quality Parameters

Phenotypic variation within the Solina landrace was already observed by Bonvicini [13], who, several decades ago, collected 53 Solina accessions and categorized them in 15 groups based on the period of earing, height plant, resistance to rust, and color of the spikes. Noteworthy is that Bonvicini selected two performing Solina lines based on rust resistance and capacity of constant production [13]. What happened to these two selected lines is not known, but it is reasonable to suppose that, in a historical period in which Italy closed the customs and depended only on its own stocks of grain, they were diffused among local farmers in order to comply with national policies that encouraged the use of varieties having the characteristics selected by Bonvicini [13]. This hypothesis is supported by the fact that, nowadays, constant production is a distinctive characteristic of the Solina landrace that grants its appreciation by local farmers [2].

Even though it was not in the purpose of this work to verify if the two different genetic clusters identified in this study resulted from an adaptation of the two lines selected by Bonvicini [13], it was of interest to investigate if phenotypic variations among Clu1 and Clu2 in terms of yield, TW, TKW, and protein content occur. Yield, TW, and protein are of utmost economical interest for local Solina producers; this is because TW and protein content are grade-determining factors for the commercial quality of wheat. Moreover, protein content is an essential criterion for breadmaking characteristics, since higher protein generally produce doughs more resistant to overworking during mixing and breads with greater loaf volume.

Clu1 that was collected in a wide area characterized by a mean altitude of 703 m showed higher TW, TKW, and protein contents than Clu2 that was collected in a restricted area characterized by a mean altitude of 883 m and located in the south-east of the sampling area. Differences in TW and TKW were observed in both the seeds set collected in 2018 and in the samples collected in the in situ experiments conducted in 2019 and 2020, whilst protein content resulted significantly different only in the in situ experiments. This result is due to the fact that the samples collected in 2018 were cultivated in different geographical areas (Figure 1), pedo-climatic conditions, and under different agronomic management conditions, thus presenting an extremely broad range of protein content. These results are in accordance with literature, in fact, protein content is reported to be heavily affected by rainfall, temperature, and soil fertility, which makes environment the most influential effect, even greater than genotype or interaction environment x genotype on protein content [32,33].

## 5. Conclusions

The results of this investigation highlighted the genetic relationships of Solina with Turkey and Middle East landraces, and confirmed its wide genetic diversity by permitting to identify two principal genetic groups with distinct origin and quality traits. These findings constitute a first step towards protection and conservation of Solina genetic heritage, but could also have implications for its agronomic management and product end-use. Further experiments (e.g., characterization of frost tolerance, vernalization, and photoperiod genes) need to be carried out in order to study the origin of this genetic differentiation, probably due to adaptation phenomena caused by environment pressures. Despite its between accessions diversity, the Solina wheat remains genetically distant from all the worldwide cultivars and landraces reported in the CIMMYT database, thus it could be also considered as a unique genetic resource for breeding programs.

## Figures and Tables

**Table 1:** Significant *p*-values from ANOVA F-tests, for each phenotypical trait analyzed from Solina samples collected in 2018 in Abruzzo.

Effect	Type	TKW	TW	Protein %
Clu1/Clu2	Fixed	0.018	0.017	ns
Farm (Clu1/Clu2)	Random	<0.001	<0.001	<0.001

**Table 2:** Significant *p*-values from ANOVA F-tests, for each trait analyzed from in situ experiment samples conducted in 2019 and 2020.

Effect	Type	TKW	TW	Protein %	Yield
Plot	Random	ns	ns	ns	ns
Year	Fixed	ns	<0.001	ns	0.011
Farm	Fixed	<0.001	ns	ns	<0.001
Clu1/Clu2	Fixed	0.003	0.044	0.003	ns

## Data Availability

The data presented in this study are available on request from the corresponding author.

## Supplementary Materials

The following supporting information can be downloaded at: https://www.mdpi.com/article/10.3390/plants11091170/s1, Figure S1: Plot of missing values (%) and heterozygosis (%) for Bulk and single Seeds dataset, Figure S2: Marker density plot for Bulk (a), Single Seed (b), and CIMMYT/Solina (c) datasets along the 21 chromosomes, Figure S3: PCA 3D score plot computed by using CIMMYT/Solina dataset (3842 markers and 24,868 genotypes of which 91 were Solina seeds). The ellipsoids indicate the 95% confidence limit for each Solina cluster. Colors indicate biological status: CIMMYT landraces (green spheres); CIMMYT cultivars (red spheres); Solina genotypes (blue spheres), Table S1: Geographical location of 24 Solina accessions collected in 2018 in Abruzzo region, Table S2: Numbers of SNP markers detected and filtered in the three datasets, also ordered per genome and chromosome.
