# Supplementary material for: Exploration of the Genetic Diversity of Solina Wheat and Its Implication for Grain Quality
Source: Plants (Basel). 2022 Apr 26;11(9):1170. doi: 10.3390/plants11091170 (PMC9102871; doi:10.3390/plants11091170)
Supplement: Supplementary file 1 [file plants-11-01170-s001.zip › Figure S2 ver2.pdf]

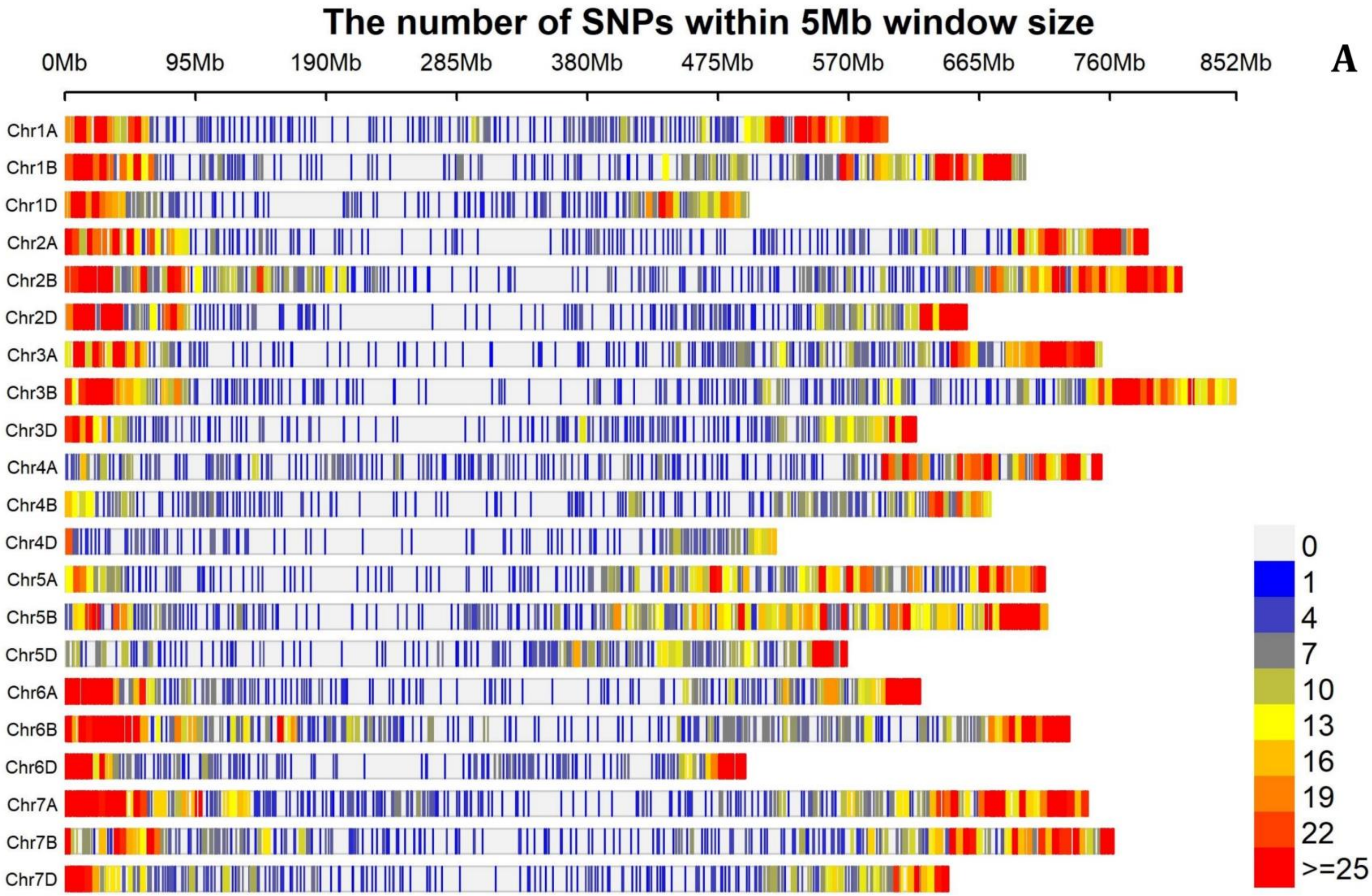

Figure S2. Marker density plot for Bulk (A), Single Seed (B) and CIMMYT/Solina (C) datasets along the 21 chromosomes.

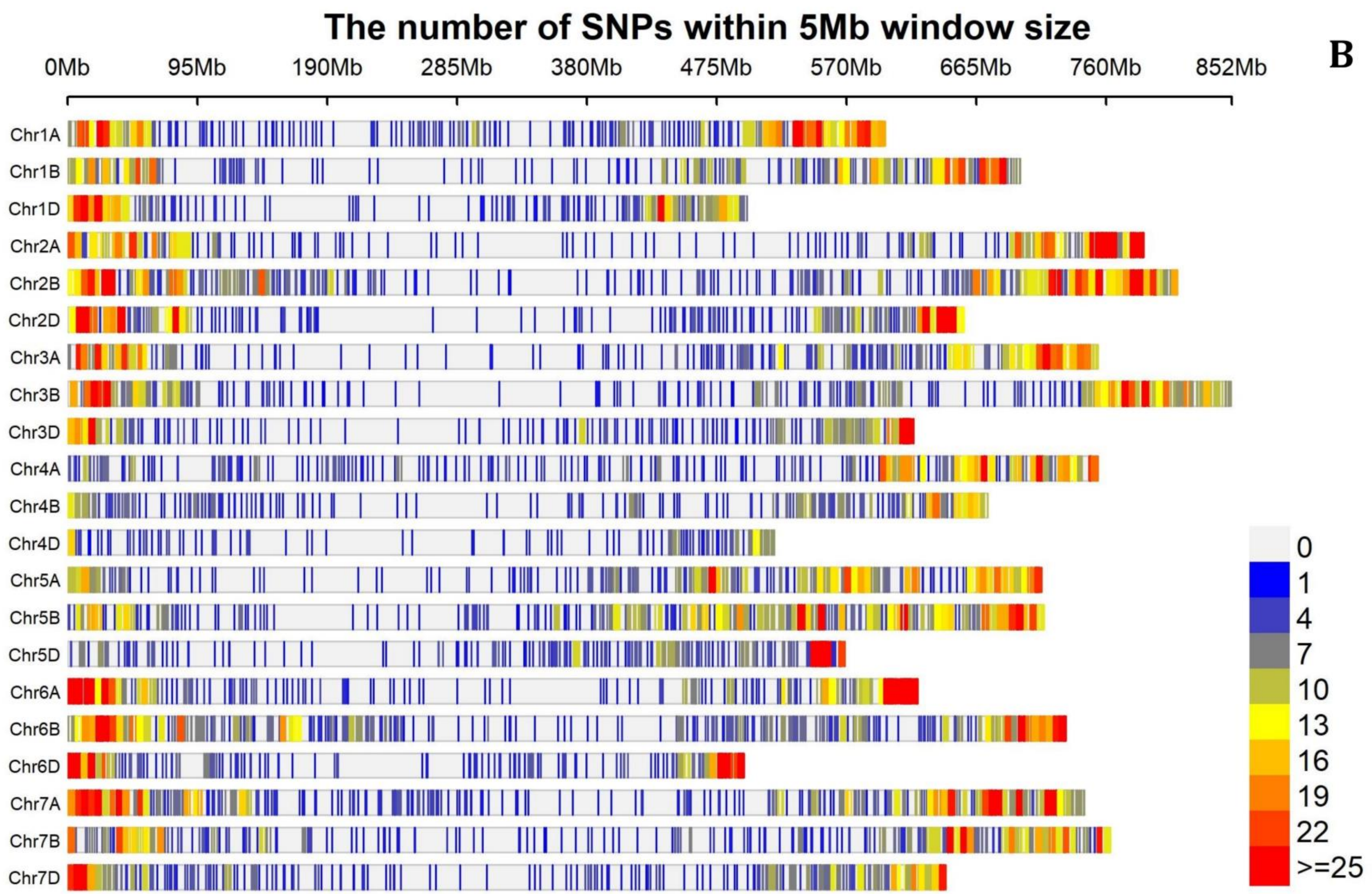

Figure S2. Marker density plot for Bulk (A), Single Seed (B) and CIMMYT/Solina (C) datasets along the 21 chromosomes.

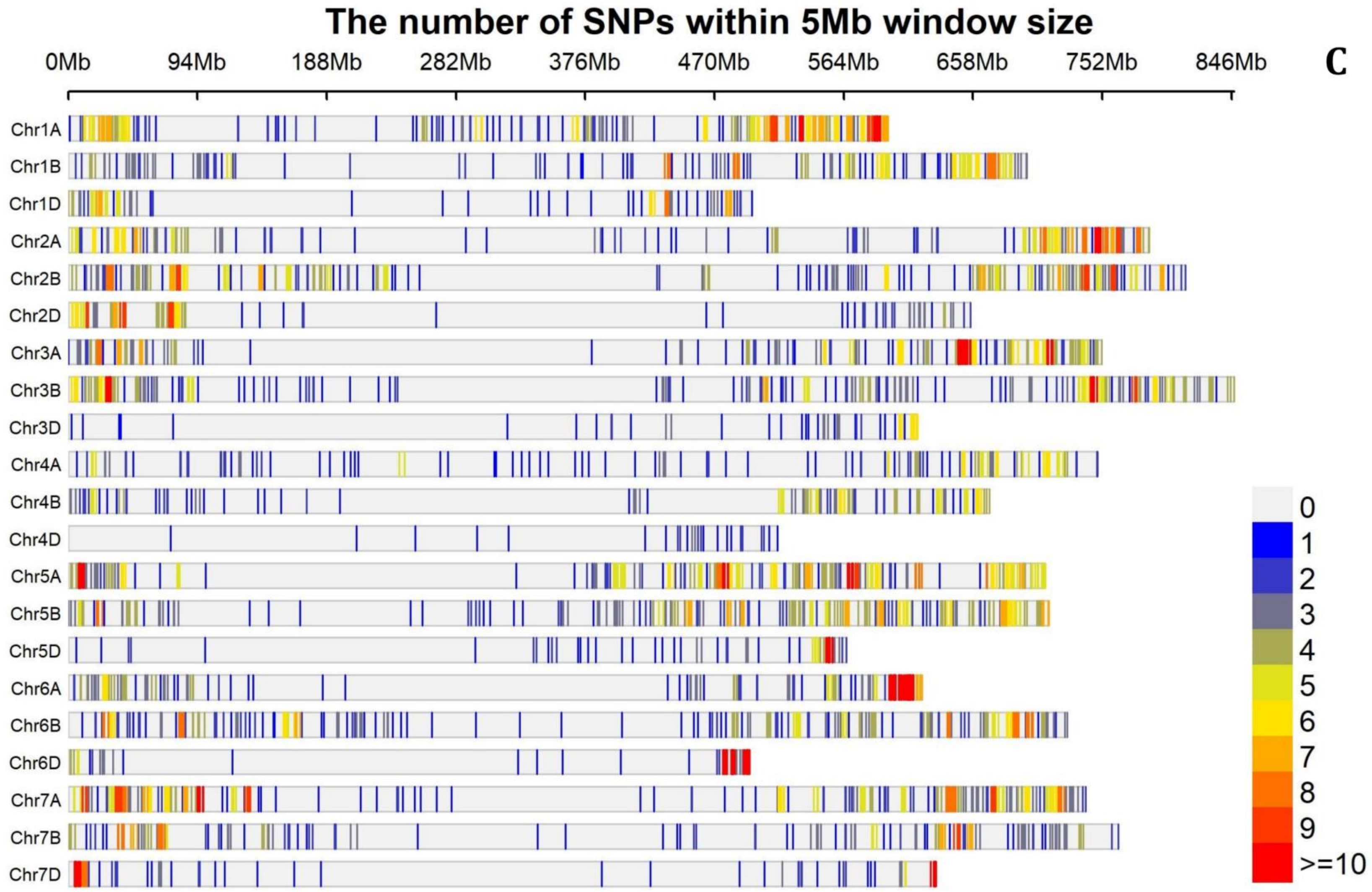

Figure S2. Marker density plot for Bulk (A), Single Seed (B) and CIMMYT/Solina (C) datasets along the 21 chromosomes.
