# Supplementary material for: Exploration of the Genetic Diversity of Solina Wheat and Its Implication for Grain Quality
Source: Plants (Basel). 2022 Apr 26;11(9):1170. doi: 10.3390/plants11091170 (PMC9102871; doi:10.3390/plants11091170)
Supplement: Supplementary file 1 [file plants-11-01170-s001.zip › Figure S3 ver2.pdf]

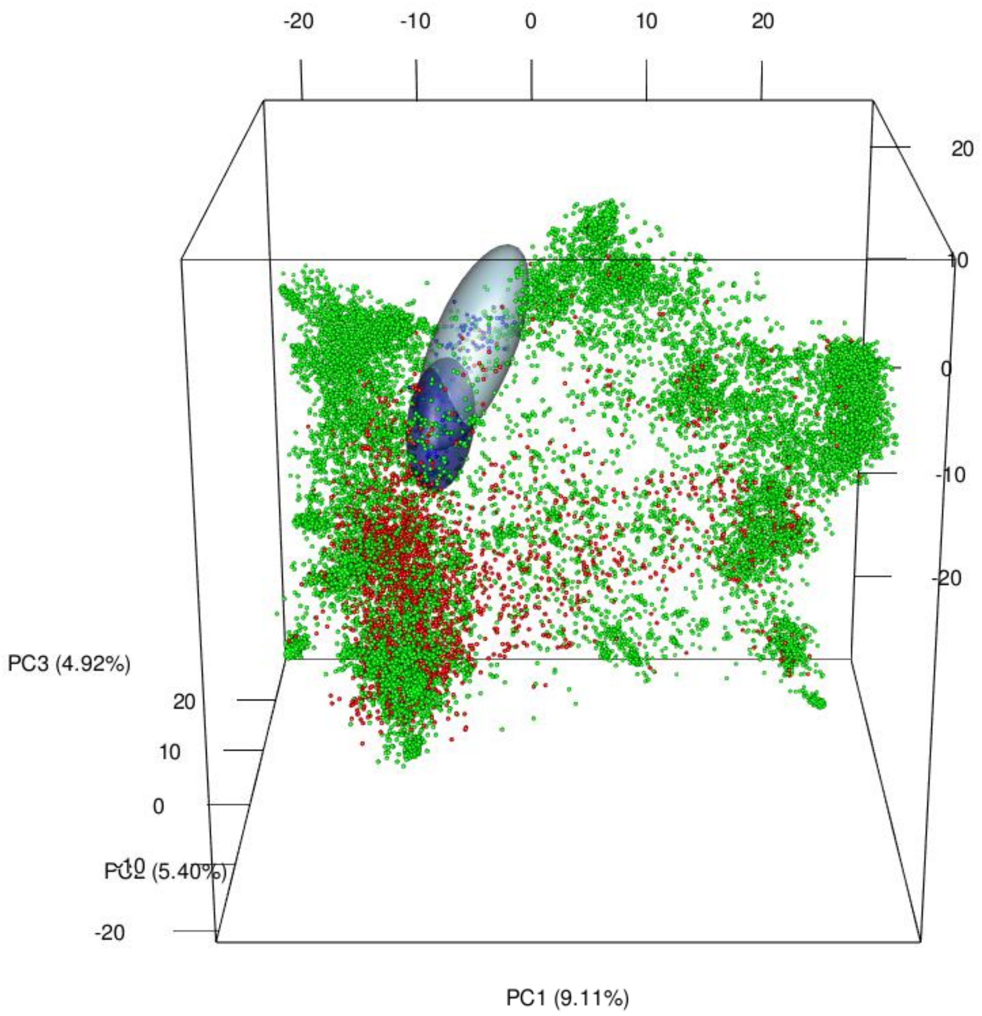

Figure S3. PCA 3D score plot computed by using CIMMYT/Solina dataset (3,842 markers and 24,868 geno-types of which 91 Solina seeds). The ellipsoids indicate the 95% confidence limit for each Solina cluster. Colours indicate biological status: CIMMYT landraces (green spheres); CIMMYT cultivars (red spheres); Solina genotypes (blue spheres).
