# Supplementary material for: Exploration of the Genetic Diversity of Solina Wheat and Its Implication for Grain Quality
Source: Plants (Basel). 2022 Apr 26;11(9):1170. doi: 10.3390/plants11091170 (PMC9102871; doi:10.3390/plants11091170)
Supplement: Supplementary file 1 [file plants-11-01170-s001.zip › Table S1.pdf]

Table S1 Geographical location of 24 Solina accessions collected in 2018 in Abruzzo region

N.ID	Location	Latitude	Longitude	Altitude (m a.s.l.)
1	Castelvecchio Subequo	42.13	13.73	492
2	Castelvecchio Subequo	42.13	13.73	492
3	Introdacqua	42.01	13.90	652
4	Scanno	41.90	13.88	1,004
5	Luco dei Marsi	41.96	13.47	664
6	Goriano Sicoli	42.08	13.77	712
7	Tagliacozzo	42.07	13.25	736
8	Rosciolo dei Marsi	42.12	13.34	902
9	Magliano dei Marsi	42.08	13.36	707
10	Magliano dei Marsi	42.08	13.36	707
11	Scurcola Marsicana	42.06	13.34	698
12	Rocca Pia	41.93	13.98	1,067
13	Scurcola Marsicana	42.06	13.34	698
14	Pescosansonesco	42.25	13.88	532
15	Farindola	42.44	13.82	514
16	Cagnano Amiterno	42.46	13.23	844
17	Castel Del Monte	42.37	13.73	1,354
18	Montereale	42.53	13.24	911
19	Capestrano	42.27	13.77	501
20	Barisciano	42.33	13.59	948
21	Capitignano	42.52	13.30	908
22	Ofena	42.33	13.76	521
23	Rivisondoli	41.87	14.07	1,309
24	Elice	42.52	13.97	249
