# Supplementary material for: Exploration of the Genetic Diversity of Solina Wheat and Its Implication for Grain Quality
Source: Plants (Basel). 2022 Apr 26;11(9):1170. doi: 10.3390/plants11091170 (PMC9102871; doi:10.3390/plants11091170)
Supplement: Supplementary file 1 [file plants-11-01170-s001.zip › Table S2.pdf]

Table S2 Numbers of SNP markers detected and filtered in the three datasets, also ordered per genome and chromosome.

	Bulk dataset	Single Seed dataset	CIMMYT/Solina dataset
1A	994	746	262
1B	1,003	657	195
1D	580	441	89
2A	1,067	728	229
2B	1,368	1,004	271
2D	844	610	108
3A	970	703	216
3B	1,108	727	245
3D	575	423	47
4A	935	659	150
4B	527	413	124
4D	259	174	25
5A	951	687	270
5B	1,217	912	270
5D	558	387	62
6A	1,018	676	166
6B	1,331	868	241
6D	669	462	61
7A	1,450	931	280
7B	1,096	698	175
7D	792	558	75
Genome A	7,385	5,130	1,573
Genome B	7,650	5,279	1,521
Genome D	4,277	3,055	467
Total filtered	23,741	15,959	3,842
