# Supplementary figures and images for: Exploration of the Genetic Diversity of Solina Wheat and Its Implication for Grain Quality
Source: Plants (Basel). 2022 Apr 26;11(9):1170. doi: 10.3390/plants11091170 (PMC9102871; doi:10.3390/plants11091170)

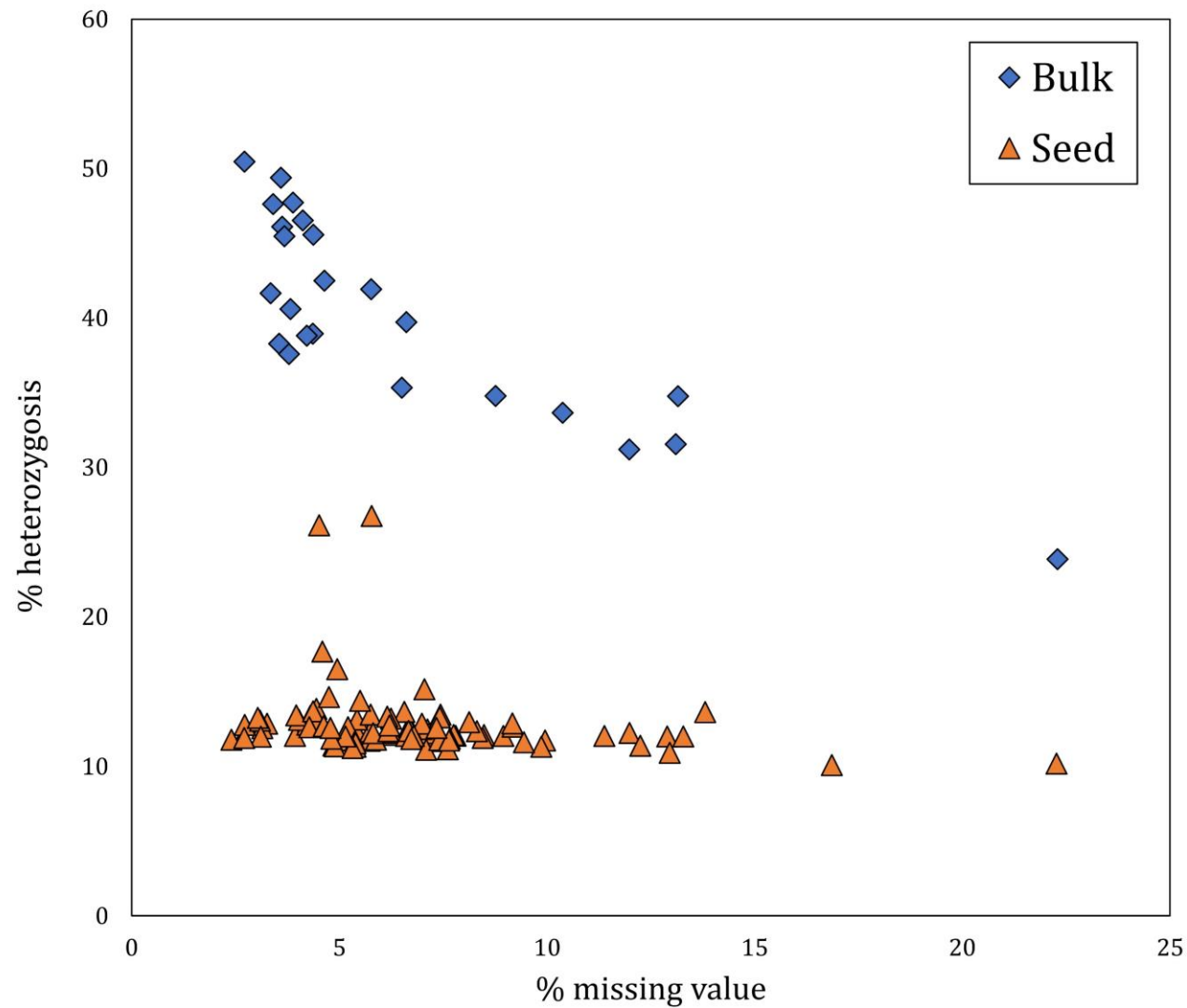

**Figure S1.** Plot of missing values (%) and heterozygosity (%) for Bulk and single Seeds dataset.

Supplement: Supplementary file 1 [file plants-11-01170-s001.zip › Figure S1 ver2.pdf]
